# Facilitators and barriers to implementing clinical care pathways

**DOI:** 10.1186/1472-6963-10-182

**Published:** 2010-06-28

**Authors:** Sara Evans-Lacko, Manuela Jarrett, Paul McCrone, Graham Thornicroft

**Affiliations:** 1 Health Service and Population Research Department, Institute of Psychiatry, King's College London, De Crespigny Park, London, SE5 8AF, UK; 2 Centre for the Economics of Mental Health, Health Service and Population Research Department, Institute of Psychiatry, De Crespigny Park, London, SE5 8AF, UK

## Abstract

**Background:**

The promotion of care pathways in the recent Governmental health policy reports of Lord Darzi is likely to increase efforts to promote the use of care pathways in the NHS. Evidence on the process of pathway implementation, however, is sparse and variations in how organisations go about the implementation process are likely to be large. This paper summarises what is known about factors which help or hinder clinicians in adopting and putting care pathways into practice, and which consequently promote or hinder the implementation of scientific evidence in clinical practice.

**Discussion:**

Care pathways can provide patients with clear expectations of their care, provide a means of measuring patient's progress, promote teamwork on a multi-disciplinary team, facilitate the use of guidelines, and may act as a basis for a payment system. In order to achieve adequate implementation, however, facilitators and barriers must be considered, planned for, and incorporated directly into the pathway with full engagement among clinical and management staff. Barriers and/or facilitators may be present at each stage of development, implementation and evaluation; and, barriers at any stage can impede successful implementation. Important considerations to be made are ensuring the inclusion of all types of staff, plans for evaluating and incorporating continuous improvements, allowing for organisational adaptations and promoting the use of multifaceted interventions.

**Summary:**

Although there is a dearth of information regarding the successful implementation of care pathways, evidence is available which may be applied when implementing a care pathway. Multifaceted interventions which incorporate all staff and facilitate organisational adaptations must be seriously considered and incorporated alongside care pathways in a continuous manner. In order to better understand the mechanism upon which care pathways are effective, however, more research specifically addressing conditions under which providers become engaged in using care pathways is needed.

## Background

Care pathways may serve as useful and evidence-based tools to reduce variations in clinical practice and improve quality and outcomes of healthcare interventions. Care pathway implementation is likely to become increasingly emphasised in England given its prominence within the recent Governmental health policy reports of Lord Darzi [1,2]. Care pathways are cited by Darzi as a form of quality improvement to be implemented in the NHS, and indeed pathway development has already begun for selected health conditions in all regions of the country [1,2].

Although definitions of what care pathways entail vary somewhat, two components consistently play a role: (a) the types of services/interventions provided; and (b) the timeline over which these happen [3]. Although several terms have been used largely synonymously (including clinical pathways and clinical care pathways) to refer to this concept, in this paper we shall refer to *care pathways *[4].

### The care pathway concept

This paper reviews the evidence on whether care pathways may ameliorate weaknesses in the implementation of guidelines and protocols by more specifically engaging with the clinical team at each stage of pathway development. In particular we aim to identify which factors facilitate or present barriers to care pathway implementation. Such care pathways incorporate clinical guidelines in that the latter are embedded into the pathway itself. Although clinical guidelines are usually developed in a top-down fashion, care pathways are more often derived from the bottom-up, so that the pathway precisely fits the configuration of the local heath service. Such pathways may adapt to nuances between institutional cultures by including teams of clinical service providers and managers in their local creation and implementation. Moreover, the process of creating a pathway calls for individuals from all sectors to be involved in defining their own roles, in terms of responsibilities and relationship to others in the local 'healthcare economy'. Consequently, there should be a sense of participation in pathway design but also accountability in terms of implementation.

## Discussion

### Engagement in developing care pathways

Successful implementation of care pathways, to a large extent, depends on the involvement and investment of both clinical service providers and managers[5]. Engagement of all relevant staff is necessary to ensure proposed aims are achieved, at each stage from pathway adoption, implementation and maintenance. Although interest in care pathways has recently and rapidly increased within the NHS [1,2], it is important to appreciate that the evidence base on pathway creation greatly exceeds what is known on how to engage providers and how to modify their practice [6]. Several studies suggest that simply providing information alone does not impact evidence-based practice [7-9]. Many healthcare institutions make such initiatives without significant clinician engagement [10]. The proposed NHS Constitution in England [11] pledges to address this by striving to "engage staff in decisions that affect them and the services they provide." Consequently, building and applying the evidence on clinical engagement will be significant toward achieving successful implementation of pathways.

The specific intent of involving a range of providers in the pathway process, is significant, but is still subject to barriers which occur in guideline implementation and therefore insufficient to ensure clinical engagement. As the literature on implementing care pathways is sparse, this paper also draws on lessons from clinical guidelines and protocols, as they have received more scrutiny, especially examining active implementation efforts rather than passive diffusion [12-14]. Specifically, we carried out a search on the databases of CINAHL 1982-2007, EMBASE 1980-2007, Ovid MEDLINE 1996-2007, Journals@Ovid Full Text June 2006, EBM CCTR, CDSR, DARE, ACP Journal Club, all 2^nd ^quarter 2007, using the search terms clinical, care, critical, integrated in combination with pathway/s; also guidelines, protocol, implementation, evaluation, facilitate$, barriers combined with the former search terms. Inclusion criteria for this review were English speaking peer reviewed articles focusing on evaluated or synthesised interventions used to implement guidelines, protocols or pathways at a system level. Two researchers (SEL and MJ) coded facilitators and barriers discerned from the literature. We do not report the results from all articles; however this reviewing strategy ensured saturation of all types of facilitators and barriers reported in the literature. Based on this review, we address potential barriers which may impede implementation and suggest interventions which may increase the uptake and usage of care pathways.

### Barriers to clinical engagement

**Figure 1:**
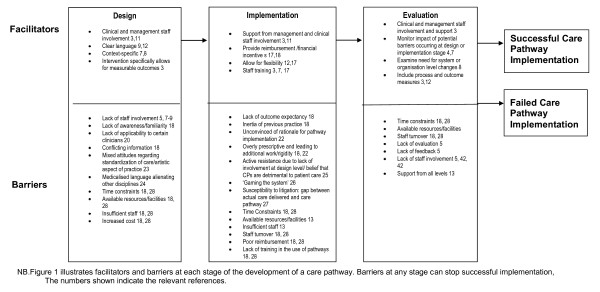
**Stages in the Development of Evidence-Based Care Pathways (CPs)**.

Barriers which impede clinical engagement and uptake of care pathways may occur at the staff (clinician or management) or healthcare organisation (management, resources, and financial or institutional structures) level or be influenced by external factors (broader health and social policies or patient characteristics) [2,5,13,15-17]. Most literature, however, focuses on clinician-related barriers. This evidence is shown in Figure 1, which illustrates both facilitators and barriers in the design, implementation and evaluation of care pathways.

A review by Cabana et al. [18], categorised barriers as those which relate to clinician knowledge, attitudes, and behaviours. Knowledge-related barriers stem from lack of awareness and familiarity [18]. Studies have demonstrated knowledge gaps among clinicians in following the publication of guidelines [19] though modifying documentation or wording may make them more user-friendly [20,21]. Cabana notes attitude-related barriers, including general guideline disagreement, lack of applicability to certain clinicians, conflicting information among different sets of guidelines, lack of outcome expectancy, and inertia of previous practice [18]. Additional attitude-related barriers may result from clinicians being unconvinced of the rationale for pathway implementation. For instance, pathways may be perceived to be driven by reasons relating to management or cost containment rather than as decision-supporting tools [22].

Clinicians may have mixed or negative attitudes regarding standardisation of healthcare through the use of pathways. For example, Jones notes that providers feel care pathways might compromise the "artistic aspect of practice" [23]. Even though clinicians may appreciate the guidance and information that pathways can provide, they may also feel that pathways are externally imposed and threaten clinical autonomy by being overly prescriptive and leading to additional work [18,22]. For example, the criticism that pathways are excessively prescriptive may be countered by the assumption that the development of healthcare tariffs, such as the Payment by Results system in England (PBR), requires some standardisation of care. For PBR to work, the tariff paid must relate to care packages that will not vary greatly between providers.

Support for care pathways may also differ by type of provider. Some studies suggest nursing staff view pathways more negatively than physicians. This may be explained, in part, by use of language which is perceived as "medicalised" and by excluding nurses or other professionals in pathway development [24]. Staff having more involvement may attach greater value to incorporating recommended practices into their behaviour, while clinicians who were not involved may perceive pathways as burdensome or detrimental to patient care, making them reluctant to adhere to the pathway as it was designed [25]. Consequently, care pathways may contribute to providers deliberately 'gaming', for example, to miscode a patient's diagnosis in order to facilitate specific interventions for particular patients [26], possibly due to beliefs that a certain treatment will not be effective due to a patient's inability or opposition to follow through with certain treatments, goals of obtaining higher reimbursement (so called 'cream-skimming'), or reluctance to put a patient into a specified pathway group. Providers may also be concerned that comparing the actual care delivered with a care pathway makes them more susceptible to litigation should the patient suffer harm during the course of treatment [27].

Although responsibility for guideline adherence often focuses on clinicians, factors external to the clinician may present significant barriers to engagement. Types of organisational or external barriers noted in the literature include time constraints, available resources/facilities, insufficient staff, staff turnover, variation in implementation across teams, poor reimbursement, lack of training in the use of pathways, increased costs (practice and liability) and patient characteristics [18,28]. A survey of 17 European countries, for example, found that the influence of external bodies (such as purchasers), lack of encouragement and financial support for pathway development, and payment incentives to be the most commonly reported organisational or external constraint to pathway implementation [29]. Less malleable factors, however, such as patient characteristics may also play a role; certain patients may be better informed, or exert their preferences for certain treatments which may or may not be recommended [30].

### Interventions to improve clinical engagement

No one implementation strategy can be expected to be successful in all contexts, and the literature suggests a multifaceted intervention which is setting-specific is most likely to be effective [18] Since barriers occur at several levels, interventions which do not focus solely on the clinician are more effective. Therefore, selecting an intervention, requires consideration of both context and persons for which it will be applied [5,31]. This may include clinical and management staff and may occur at the Trust (provider organisation) or unit level. Greenhalgh noted seven key areas of consideration which must be evaluated separately for each organisation when introducing any type of systemic change(1) characteristics of the innovation itself, (2) characteristics of the individuals targeted to adopt it, (3) sources of communication and influence regarding the innovation, (4) structural and cultural characteristics of targeted organisations (5) external influences on targeted individuals or organisations, (6) organisations' uptake processes, and (7) the inter-linkages among these six factors [13].

Organisational culture and characteristics provide context for understanding and choosing the most effective mechanism of change [16]. There are, however, several options of interventions which might be tailored to medical care settings. Grimshaw et al., identified six types of published interventions: broad strategies, dissemination and implementation of guidelines, programs to enhance the quality and economy of primary care, interventions to improve doctor-nurse collaboration, targeting of specific behaviours, and focusing on the effectiveness of specific interventions [6]. Rather than advocating a specific intervention, Grimshaw et al., concluded that effectiveness depends on context and, in general, passive approaches are unlikely to affect behaviour [6]. A review of quality improvement interventions supported this showing that no intervention seemed to be associated with large improvement and only multifaceted interventions were associated with even moderate levels of improvement [17].

Evaluations of specific types of interventions showed a wide range of effectiveness, suggesting again that the context and implementation process may be as important as the intervention itself [32]. Therefore, although active, multiple and co-ordinated interventions may be costly, there is evidence that they are often more effective too. In an examination of failed interventions, a combination of "continued reliance on passive diffusion", disagreement regarding guideline content, provider characteristics, and logistic or financial barriers were hypothesised as the reasons most often explaining failure to change behaviour [17]. Methodological evaluation, however, may also be complicated by lack of information regarding baseline adherence [18], and different combinations of interventions.

Although the literature emphasises a multi-faceted approach, strengths and weaknesses of specific interventions have also been analysed. One common intervention used to change provider behaviour is Continuing Medical Education (CME). Though traditional CME didactic lectures and conferences have been shown to be ineffective at changing behaviour, use of several targeted and sequenced activities may have a positive impact [33-36]. Still, little is known about the most effective way to implement training programs for providers [35]. Moreover, it seems that, as with many interventions, more effective methods are often relatively costly and are rarely implemented [6].

Decision aid tools have been tested as a way to improve provider knowledge and streamline provider behaviour. Overall, most trials show improved provider performance in a variety of settings, though information regarding patient outcomes is lacking [37,38]. An RCT of decision support for treatment of depression vs. usual care found process of care (e.g., ongoing specialty care and psychotropic treatment) and patient satisfaction improved, although clinical outcomes did not [39].

Change in provider behaviour may require system level change. Although some organisational factors are less malleable than others, (e.g., resource availability), more flexible considerations such as staff restructuring might be implemented to accommodate care pathways [40]. Current care processes should also be evaluated in terms of their consistency with the implemented pathway and ongoing feedback from staff should inform where systemic barriers lie. Finally, alignment of payment with interventions may improve uptake and subsequent practice [35], in order to act as an incentive for providers to 'comply' with care pathways that have determined the tariff used in commissioning services. Financial incentives have previously been shown to be useful ways of encouraging change, for example in relation to GPs achieving Quality and Outcomes Framework targets such as assessing levels of depression among their caseload^41^.

## Conclusion

Care pathways may have merit in terms of their potential to improve healthcare practice^3^. They can focus resources, provide a clear understanding for patients of what they should expect in their care, and provide a means of measuring patient's progress. They promote teamwork via increased understanding of roles on a multi-disciplinary team, and facilitate the use of guidelines in clinical practice in a usable format [20,41]. Furthermore, they may act as a basis for a prospective payment system, such as PBR. In order to achieve adequate implementation, however, serious consideration of potential barriers and specific locally agreed upon interventions to ensure effective implementation must be planned for and incorporated directly with the pathway. As with any quality improvement intervention, implementation and evaluation are a continuous process. This process needs to involve all those taking part in the protocol and following identification and dissemination, there must be decision support available, evaluation of the application, and finally incorporation of results into ongoing quality improvement [5,41,42]. Successful implementation of care pathways is dependent on the development process. A lack of understanding about their role and use by any staff group will doom them to failure [41]. In order to better understand the mechanism upon which care pathways are effective, more research specifically addressing conditions under which providers become engaged in using care pathways is needed.

## Summary

• The promotion of care pathways in the Darzi reports is likely to increase efforts to promote the use of care pathways in the NHS.

• Inclusion and accountability of both clinical and management staff (at both junior and senior levels) in pathway development and implementation is necessary for the successful uptake of care pathways.

• Evidence on the process of pathway implementation is sparse and variations in how organisations go about the implementation process are likely to be large.

• Multifaceted interventions which involve changing provider behaviour in addition to organisational adaptations must be seriously considered and incorporated alongside care pathways in a continuous manner.

## Competing interest Statement

All authors were staff of the London Mental Health Clinical Care Pathway Group of which GT was a co-chair. This article arose from discussion of work done in the group and in response to the recommendations in the report: *Healthcare for London: A Framework for Action*. M J, S E-L, and PM received financial support from NHS London in conducting a literature review that supported the writing of this paper. This study was funded in relation to the NIHR Specialist Mental Health Biomedical Research Centre at the Institute of Psychiatry, King's College London and the South London and Maudsley NHS Foundation Trust (GT). GT and PM are supported in relation to a National Institute for Health Research (NIHR) Applied Programme grant awarded to the South London and Maudsley NHS Foundation Trust, and in relation to the NIHR Specialist Mental Health Biomedical Research Centre at the Institute of Psychiatry, King's College London and the South London and Maudsley NHS Foundation Trust.

## Authors' contributions

SEL participated in the conception and design of the study, drafting the article and revising it critically for important intellectual content, and final approval of the version to be published. MJ participated in the conception and design of the study, drafting the article or revising it critically for important intellectual content, and final approval of the version to be published. PM participated in the conception and design of the study, drafting the article or revising it critically for important intellectual content, and final approval of the version to be published. GT participated in the conception and design of the study, drafting the article or revising it critically for important intellectual content, and final approval of the version to be published.

## Authors' Information

SEL is a postdoctoral research fellow, MJ is a research worker and PM is a reader in health economics at the Institute of Psychiatry, King's College London. GT is Professor of Community Psychiatry and Head of the Health Service and Population Research Department at the Institute of Psychiatry, King's College London. He is a Consultant Psychiatrist and Director of Research and Development at the South London and Maudsley NHS Foundation Trust. All authors participated in the drafting and editing of the article and approved the final version.

## Pre-publication history

The pre-publication history for this paper can be accessed here:

http://www.biomedcentral.com/1472-6963/10/182/prepub
